# Mycobacterial P_1_-Type ATPases Mediate Resistance to Zinc Poisoning in Human Macrophages

**DOI:** 10.1016/j.chom.2011.08.006

**Published:** 2011-09-15

**Authors:** Hélène Botella, Pascale Peyron, Florence Levillain, Renaud Poincloux, Yannick Poquet, Irène Brandli, Chuan Wang, Ludovic Tailleux, Sylvain Tilleul, Guillaume M. Charrière, Simon J. Waddell, Maria Foti, Geanncarlo Lugo-Villarino, Qian Gao, Isabelle Maridonneau-Parini, Philip D. Butcher, Paola Ricciardi Castagnoli, Brigitte Gicquel, Chantal de Chastellier, Olivier Neyrolles

**Affiliations:** 1 Centre National de la Recherche Scientifique, Institut de Pharmacologie et de Biologie Structurale, 31000 Toulouse, France; 2 Université de Toulouse, Université Paul Sabatier, Institut de Pharmacologie et de Biologie Structurale, 31000 Toulouse, France; 3 Aix Marseille Université, Faculté des Sciences de Luminy, Centre d'Immunologie de Marseille-Luminy, F-13288 Marseille, France; 4 Inserm, U631, Centre d'Immunologie de Marseille-Luminy, F-13288 Marseille, France; 5 CNRS, UMR6102, Centre d'Immunologie de Marseille-Luminy, F-13288 Marseille, France; 6 Key Laboratory of Medical Molecular Virology, Fudan University, 200032 Shanghai, China; 7 Institut Pasteur, Unité de Génétique Mycobactérienne, 75015 Paris, France; 8 Brighton and Sussex Medical School, University of Sussex, Brighton BN1 9PX, UK; 9 Department of Biotechnology and Bioscience, University of Milano-Bicocca, 20126 Milan, Italy; 10 Centre for Infection, Division of Clinical Sciences, St. George's University of London, London SW17 0RE, UK; 11 Singapore Immunology Network, 138648 Singapore, Singapore

## Abstract

*Mycobacterium tuberculosis* thrives within macrophages by residing in phagosomes and preventing them from maturing and fusing with lysosomes. A parallel transcriptional survey of intracellular mycobacteria and their host macrophages revealed signatures of heavy metal poisoning. In particular, mycobacterial genes encoding heavy metal efflux P-type ATPases CtpC, CtpG, and CtpV, and host cell metallothioneins and zinc exporter ZnT1, were induced during infection. Consistent with this pattern of gene modulation, we observed a burst of free zinc inside macrophages, and intraphagosomal zinc accumulation within a few hours postinfection. Zinc exposure led to rapid CtpC induction, and *ctpC* deficiency caused zinc retention within the mycobacterial cytoplasm, leading to impaired intracellular growth of the bacilli. Thus, the use of P_1_-type ATPases represents a *M. tuberculosis* strategy to neutralize the toxic effects of zinc in macrophages. We propose that heavy metal toxicity and its counteraction might represent yet another chapter in the host-microbe arms race.

## Highlights

► Zinc accumulates in the *M. tuberculosis* (*Mtb*) phagosome in macrophages (Mϕ) ► *Mtb* P_1_-type ATPases, including CtpC, are induced upon exposure to zinc inside Mϕ ► CtpC enables *Mtb* resistance to zinc poisoning and intracellular survival in Mϕ ► P_1_-type zinc efflux ATPase ZntA null *E. coli* is highly susceptible to Mϕ killing

## Introduction

Macrophages and other phagocytes use a diverse arsenal of subcellular and molecular weapons to clear intracellular microbes. One strategy is to directly expose the invading pathogen to a toxic intracellular environment. For example, they make use of the fusion of microbial vacuoles, or phagosomes, with acidic and hydrolase-rich lysosomes, in combination with the production of antimicrobial peptides, and toxic reactive oxygen and nitrogen species (Pluddemann et al., 2011). Another strategy involves the sequestration of nutrients important for microbial growth. For instance, they may use intracellular solute transporters, such as the natural resistance-associated membrane protein (NRAMP)1/SLC11A1, NRAMP2/DMT1/SLC11A2, and ferroportin FPN1/SLC40A1, located at the phagosome membrane, and soluble chelators such as lactoferrin, which is found in the phagosome lumen, to deplete the microbial vacuoles of iron and possibly of other important nutrients, thereby impairing the intracellular development of the microbe (Cellier et al., 2007; Vidal et al., 1993). Known as the NRAMP model, the latter strategy is thought to be the current paradigm, with the depletion of important metals other than iron, such as zinc and manganese, as its main tenet and as an obvious feature of the innate immune arsenal of higher organisms (Appelberg, 2006; Kehl-Fie and Skaar, 2010).

The tuberculosis bacillus, *Mycobacterium tuberculosis*, is one of the well-known intracellular pathogens to thrive inside macrophages. It does so by interfering with the intracellular endocytosis machinery, blocking phagosome-lysosome fusion, thereby avoiding the cytolytic environment of a phagolysosome, and multiplying within macrophages (Armstrong and Hart, 1971; de Chastellier, 2009; Rohde et al., 2007). Several mechanisms involved in the intracellular survival of pathogenic mycobacteria have been identified in target-based and system-wide approaches. For example, *M. tuberculosis* may use the superoxide dismutase SodA to withstand the phagocytic oxidative burst (Edwards et al., 2001), the serine protease Rv3671c may protect against acid exposure (Vandal et al., 2008), and mycobactins and other iron transport systems may overcome iron sequestration mechanisms (De Voss et al., 2000; Rodriguez and Smith, 2006; Siegrist et al., 2009) within macrophages.

To further understand the strategies employed by *M. tuberculosis* to instigate, subvert, or disrupt antimicrobial functions in human macrophages, we previously carried out transcriptomic studies, in parallel, on both intracellular mycobacteria and their host macrophages during the infection process (Tailleux et al., 2008). Surprisingly, we found signatures of heavy metal poisoning in intracellular mycobacteria, including induction of the putative heavy metal efflux P_1_-type ATPase CtpC (Rv3270). P-type ATPases form part of a large family of ion pumps that are present in almost all kingdoms of life, including nonpathogenic species of *Mycobacterium* (Chan et al., 2010). These proteins are characterized by an autocatalytic phosphorylation step (hence the name of these transporters) and are classified into subgroups according to the ion transported (Arguello et al., 2007; Chan et al., 2010). The *M. tuberculosis* genome (Cole et al., 1998) indeed encodes 12 putative transporters of this type: seven P_1B_-type heavy metal-transporting ATPases (CtpA-D, CtpG, CtpJ, and CtpV); one P_1A_-type K^+^ transporter, KdpB; one P_2A_-type Ca^2+^ or Mg^2+^ transporter, CtpF; and three unusual P-type ATPases, CtpE, CtpH, and CtpI. Members of the P_1B_ class are mostly involved in heavy metal efflux and detoxification (Arguello et al., 2007; Chan et al., 2010), and the induction of CtpC, CtpG, and CtpV, which belong to this class of P-type ATPases, in intracellular mycobacteria suggested that the bacilli were facing an excess of free metal inside host cells. In an earlier study, heavy metals, namely zinc and copper, were found to accumulate in phagosomes containing various mycobacterial species; interestingly, zinc accumulation was even furthered upon macrophage activation by inflammatory signals such as IFN-γ and TNF (Wagner et al., 2005). In light of these previously published findings, we closely re-examined our macrophage transcriptome during *M. tuberculosis* infection (Tailleux et al., 2008) and found transcriptional signatures of heavy metal overload in the host cell, such as induction of zinc resistance genes.

Here we now report that *ctpC* is a zinc-responsive gene in *M. tuberculosis* that is likely involved in zinc efflux given that our *ctpC* null mutant was hypersusceptible to physiological concentrations of zinc and exhibited an impaired growth in human macrophages. By using a combination of fluorescence and electron microscopy-based approaches, we visualized an early burst of free zinc in host cell endocytic compartments during infection, as well as in the mycobacterial vacuole. Furthermore, free zinc also accumulated in phagolysosomes containing nonpathogenic microbes, such as *Escherichia coli*. General ablation of the zinc efflux system in *E. coli* led to faster intracellular killing of this microbe. Therefore, in addition to nutrient depletion, macrophages may make use of heavy metal (zinc) poisoning as a mechanism of antimicrobial immunity. At the same time, our study proposes the use of P_1_-type ATPases as a strategy for *M. tuberculosis* and other microbes to counteract the toxic effects of zinc in macrophages, thereby suggesting that genes such as *ctpC* might be useful targets for the treatment of tuberculosis and other diseases.

## Results

### Detection of Transcriptional Signatures Characteristic of Heavy Metal Poisoning in Both Intracellular Mycobacteria and Their Host Macrophages

**Figure 1:**
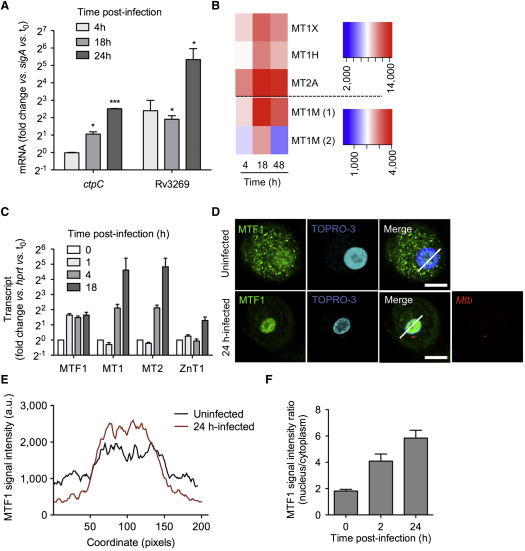
The Microbial and Host Macrophage Transcriptomes Reflect Free Zinc Overload during *M. tuberculosis* Infection (A) RT-qPCR analysis of *ctpC* and Rv3269 expression during human macrophage infection. Cells were infected for the indicated time. The data shown are means ±SD of *ctpC* expression, normalized with respect to *sigA*, in intracellular bacteria, relative to the inoculum (0). The data shown are from an experiment performed in triplicate and were analyzed with Student's t test. ^∗^p < 0.05; ^∗∗^p < 0.01; ^∗∗∗^p < 0.001; the data are representative of four independent biological replicates. (B) Transcriptional profile analysis. Red-blue density display showing the expression levels of the human metallothionein-encoding genes *MT1H*, *MT1M*, *MT1X*, and *MT2A* at 4, 18, or 48 hr after *M. tuberculosis* infection in macrophages, as reported in microarray analysis by Tailleux et al. (Tailleux et al., 2008). Genes are ordered in rows, conditions as columns. The colors indicate the strength of expression, running from red (high levels of expression) to blue (low levels of expression; in arbitrary units). (C) RT-qPCR analysis of the expression ratio of the genes encoding the metal transcription factor-1 (MTF1), MT1, MT2, and the zinc exporter ZnT1/SLC30A1 in human macrophages during *M. tuberculosis* infection, relative to uninfected cells (0). After indicated time of infection, total cellular RNA was extracted and analyzed by RT-qPCR. Data are displayed as fold-change relative to expression before infection, and are normalized relative to *hprt*. Averaged data (mean ±SD from a triplicate experiment) from one representative donor out of four tested are shown. (D–F) MTF1 localization. (D) MTF1 was immunolocalized (green) in uninfected macrophages (upper panels) and in macrophages infected as in (A) for 24 hr with DsRed-expressing *M. tuberculosis* (red, lower panels). Cell nuclei are labeled with the fluorescent dye TOPRO-3 (blue). The oblique bars indicate analysis lines as used in (E) to quantify the MTF1 signal. (E) Average MTF1 signal intensity, analyzed as in (D), for 25 cells per condition. (F) MTF1 signal intensity ratio (nucleus/cytoplasm) measured before infection (0) and 2 or 24 hr after infection. Scale white bar, 20 μm. The data shown in (D)–(F) are representative of two independent experiments, and data shown in (E) and (F) represent mean ±SD of values calculated from 40 randomly chosen cells in several fields.

Our recent transcriptome analysis of intracellular *M. tuberculosis* (Tailleux et al., 2008) revealed an early, strong, and sustained induction of the putative P_1B_-type ATPase-encoding genes *ctpC/Rv3270*, *ctpG/Rv1992c*, *ctpV/Rv0969*, and P_2A_-type *ctpF/Rv1997* during infection of human macrophages (see Figure S1A available online). Given that one of the central features of *M. tuberculosis* pathogenesis is the ability to enter and replicate inside macrophages despite encountering a toxic environment, we became interested in the possible role of P-type ATPase-encoding genes in enhancing the bacilli's fitness in host cells, all the more as the mycobacterial phagosome accumulates zinc and copper inside host macrophages (Wagner et al., 2005). Interestingly, CtpC and CtpG are phylogenetically related to zinc transporters (Figure S1B), whereas CtpV is closer to copper transporters. In addition, P_1_-type ATPase-encoding genes sometimes cluster with putative metal chaperone- and metal-responsive transcription factor-encoding genes (Figure S1C). In particular, the homologous *Rv3269*, *Rv1993c*, and *Rv0968* genes encode putative chaperones that may enhance the functional capacity of these P_1B_-ATPases, as in the case, for example, of copper delivery to copper transport ATPases (Gonzalez-Guerrero et al., 2009). Our microarray data not only revealed a strong induction of these putative chaperones during phagocyte infection but also that of the *cmtR*/*Rv1994c* and *csoR*/*Rv0967* genes known to encode metal-responsive transcriptional regulators (Figure S1D) (Cavet et al., 2003; Liu et al., 2007). Altogether, this transcriptional survey in intracellular mycobacteria hinted at a particular role for P_1B_-type ATPases and their putative partners during its establishment inside human macrophages. In particular, induction of the *ctpC*-*Rv3269* gene tandem in intracellular mycobacteria became an obvious and reproducible signature in different independent infection experiments (Figure 1A).

Assuming that *M. tuberculosis* CtpC acts as a predicted zinc efflux pump (Figure S1B), and with the knowledge that zinc was found to accumulate in the mycobacterial phagosome (Wagner et al., 2005), we hypothesized that the excess of this free metal would be reflected in the transcriptome of the host cells as well. Indeed, several metallothionein (MT)-encoding genes—*MT1H*, *MT1M*, *MT1X*, and *MT2A*—were induced in the cells during *M. tuberculosis* infection (Figure 1B). MTs are modulated in a number of stress situations, particularly those in which the cell is exposed to potentially toxic concentrations of free zinc (Palmiter, 2004). The mechanisms involved in free zinc detoxification in eukaryotic cells are well described, and include the translocation of metal-regulatory transcription-factor 1 (MTF1) to the nucleus, where it binds metal-responsive elements in the promoter regions of several genes encoding zinc detoxification proteins, including MTs and the plasma membrane zinc exporter ZnT1/SLC30A1 (Andrews, 2001; Lichten and Cousins, 2009; Murakami and Hirano, 2008). RT-qPCR analysis of *M. tuberculosis*-infected macrophages confirmed that MTs, together with MTF1 and ZnT1, were all induced (Figure 1C). In addition, immunofluorescence microscopy further demonstrated that MTF1 translocated to the macrophage nucleus upon *M. tuberculosis* infection, indicating a direct and rapid establishment of elements to withstand zinc intoxication (Figures 1D–1F). Taken together, the observed modulation of genes encoding for eukaryotic zinc detoxification proteins suggests the involvement of zinc poisoning during the antimycobacterial immune response, and confirm and expand upon previously published results (Wagner et al., 2005).

### Zn^2+^ Is Rapidly Released from Intracellular Stores upon *M. tuberculosis* Infection and Reaches the Mycobacterial Phagosome

**Figure 2:**
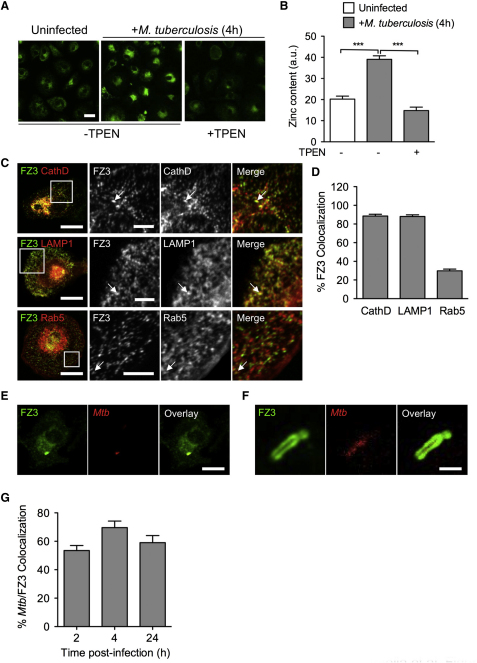
Free Zinc Is Released within Macrophages during *M. tuberculosis* Infection and Accumulates within the Mycobacterial Phagosome (A) Free zinc labeling in macrophages. Human monocyte-derived macrophages were left uninfected (left panel) or infected for 4 hr as in Figure 1A (middle and right panels), in the absence (left and middle panels) or presence (right panel) of the zinc-chelating agent TPEN. Cells were fixed and stained with the free zinc-specific fluorescent probe FluoZin-3 (FZ3, green). Scale bar, 20 μm. The picture is representative of five independent experiments. (B) FZ3 signal quantification (in arbitrary units) from ∼25 blind-scored cells from three random fields as in (A). The data shown are means ±SD of the signal measured from 20 cells and were analyzed with Student's t test. ^∗∗∗^p < 0.001; the data are representative of five independent experiments. (C) Subcellular localization of the FZ3-positive compartments in macrophages. Macrophages were transfected with RFP-reporter plasmids expressing cathepsin D (CathD, top row panels) and LAMP1 (middle row panels), or Rab5 (bottom row panels). After 4 hr of infection with *M. tuberculosis*, the cells were fixed, stained with FZ3 as in (A), and analyzed by confocal microscopy. Arrows indicate colocalization of the red and green signals. Scale bar is either 20 μm (left column panels) or 5 μm (all panels in the columns to the right). The squared fields depicted in the panels of the left column represent the magnified area shown for all panels in the columns to the right. Representative images from two experiments are shown; images are quantified in (D). (D) Quantification of FZ3 colocalization with RFP-fused CathD, LAMP1, or Rab5, as shown in (C). Percentages of colocalization (means ±SD) were determined by analyzing the proportions of FZ3-positive structures stained for CathD, LAMP1, or Rab5 from high-resolution confocal microscopy pictures. At least 1000 structures from ten cells were counted per condition to calculate mean ±SD. FluoZin-3 colocalization; the data are representative of two independent experiments. (E and F) FZ3 staining of the mycobacterial phagosome. One macrophage infected with DsRed-expressing *M. tuberculosis* (red), fixed, and stained with FZ3 is shown (E), together with a higher magnification of the mycobacterial vacuole in another macrophage (F). Scale bar is either 10 μm (E) or 1 μm (F). Representative pictures from five independent experiments are shown; images are quantified in (G). (G) Quantification of FZ3 colocalization with DsRed-expressing *M. tuberculosis* after 2, 4, and 24 hr infection. The mean percentage (±SD) of FZ3-positive phagosomes (as assessed for ∼50 phagosomes in five different fields) is shown; data are representative of three independent experiments.

The possible involvement of zinc poisoning during *M. tuberculosis* infection led us to hypothesize a possible uptake or release of excess free zinc within the infected cells, some of which may target specifically this intracellular pathogen. To test this hypothesis, we set up to visualize the presence and movement of free zinc during the *M. tuberculosis* infection by staining human macrophages with FluoZin-3 (FZ3), a fluorescent probe specific for free zinc (Domaille et al., 2008; Gee et al., 2002). Confocal microscopy showed that *M. tuberculosis* infection resulted in a rapid increase in the FZ3 signal, which was inhibited by the zinc-chelating cell-permeant agent TPEN (Figures 2A and 2B). The FZ3 signal was unaffected by chelators of other metals, such as iron, calcium, magnesium, copper, and manganese, providing additional evidence for the specificity of FZ3 (Figure S2A). In addition, the FZ3 signal was not inhibited by DTPA (Figure S2A), a cell-impermeant zinc chelator, or in the absence of zinc when cells were infected in zinc-free buffers, such as PBS. Thus, the burst of free zinc observed during infection was derived from an intracellular storage compartment rather than from the import of extracellular metal. This is not too surprising, given that zinc is mostly complexed with MTs in eukaryotic cells (Lichten and Cousins, 2009). Several stresses, such as oxygen intermediates, can dissociate zinc from MTs, resulting in the intracellular release of this metal (Andrews, 2000; Dalton et al., 1996). In *M. tuberculosis*-infected macrophages, the FZ3 signal was inhibited by apocynin, an inhibitor of the NADPH phagocyte oxidase (Figure S2B), strongly suggesting that the oxidative burst observed during mycobacterial infection leads to the dissociation of zinc from intracellular Zn^2+^-MT complexes. FZ3 staining of macrophages expressing red fluorescent reporters specific for various subcellular compartments further revealed that free zinc was released mostly in late endosomes and lysosomes, as shown by the strong colocalization of the FZ3 signal with the late endosomal markers cathepsin D (88.5% ± 5.8%) and LAMP1 (88.1% ± 5.8%) and, to a lesser extent, in early compartments of the endocytic pathway, as shown by the colocalization (29.9% ± 5.9%) of the FZ3 signal with the early endosomal marker Rab5 (Figures 2C and 2D). Close examination of mycobacterial phagosomes by confocal microscopy revealed strong zinc signals surrounding mycobacteria (Figures 2E–2G), probably corresponding to free zinc delivered to the mycobacterial vacuoles through phagosome-early endosome fusion. While it is already known that host cells alter zinc levels in response to microbial products (Kitamura et al., 2006), these results demonstrate the transport of intracellular zinc into the endosomal-lysosomal pathway in correlation to the direct exposure to an invading microbe.

### CtpC Protects *M. tuberculosis* from Excess Free Zn^2+^ and Contributes to Intracellular Survival

**Figure 3:**
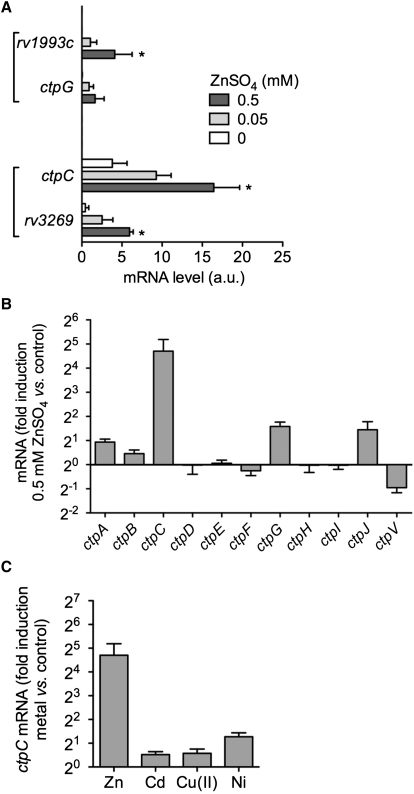
Expression of the Gene Encoding the *M. tuberculosis* Metal Cation-Transporting P-Type ATPase CtpC Is Induced by Zinc (A) Transcriptional profile analysis. mRNA levels (in arbitrary units) of the genes most strongly induced by zinc treatment in *M. tuberculosis* as revealed by microarray analysis. Bacteria were incubated with 0, 50, or 500 μM ZnSO_4_ in Sauton medium for 4 hr. Bacterial RNA was prepared for subsequent microarray analysis. The data shown are means ±SD of duplicate experiments and were analyzed with Student's t test; ^∗^p < 0.05. (B) RT-qPCR analysis of the expression of the *M. tuberculosis ctp* genes upon exposure to zinc. Bacteria were incubated with 0 or 500 μM ZnSO_4_, and RNA extracted and treated as in (A). The data shown are mean ±SD of a triplicate experiment measuring *ctp* expression, normalized with respect to *sigA*, in zinc-treated bacteria, relative to untreated bacteria. Data are representative of at least two independent experiments. (C) RT-qPCR analysis of *ctpC* expression following the exposure of *M. tuberculosis* to various divalent metal cations. Bacteria were left untreated or were incubated with 500 μM ZnSO_4_, 200 nM CdSO_4_, 500 μM CuSO_4_, or 200 μM NiSO_4_ in Sauton medium for 4 hr. The data are shown as in (B) and are representative of two independent experiments.

Based on the rapid and sustained colocalization of intracellular zinc and mycobacteria within macrophages, we investigated whether one or more of the P-type ATPases induced in intracellular mycobacteria (Figure S1A) would be specifically responsive to zinc. We performed a transcriptome analysis of *M. tuberculosis* exposed to different physiological concentrations of zinc. This transcriptional survey clearly revealed two zinc-responsive genetic modules containing P-type ATPase-encoding genes, namely *ctpG-Rv1993c* and *Rv3269-ctpC* (Figure 3A and Table S1). Of these, we were able to confirm the modulation of the *ctp* genes in response to zinc by RT-qPCR analysis. Accordingly, *ctpC* expression was strongly (up to ∼30 times) induced upon exposure to zinc (Figure 3B), but not upon treatment with other divalent cations, such as Cd^2+^, Cu^2+^, and Ni^2+^ (Figure 3C). In addition, the expression for *ctpG* and *ctpJ* was also induced, but to a lower level compared to that of *ctpC* (Figure 3B). These results indicate that the direct exposure of *M. tuberculosis* to the burst of intracellular zinc during macrophage infection might be responsible for the observed upregulation of P_1_-ATPases and their putative functional partners, particularly that of *ctpC* together with its chaperone-encoding gene *Rv3269*, thus establishing precedence for a possible cooperative role between these two genes to resist zinc intoxication.

**Figure 4:**
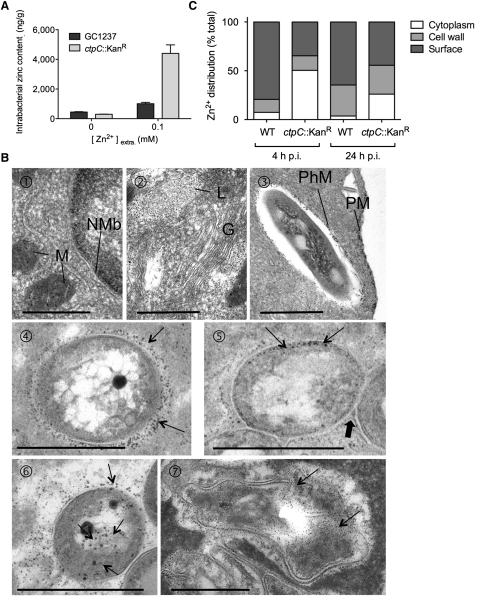
CtpC Is Involved in Zinc Efflux in *M. tuberculosis* (A) Intrabacterial zinc content in *M. tuberculosis* wild-type and a *ctpC* null mutant. *M. tuberculosis* wild-type (GC1237) or a *ctpC* null mutant (*ctpC*::Kan^R^) was left untreated or incubated with 0.1 mM ZnSO_4_ for 1 hr at 37°C, washed twice in PBS, heat inactivated, and the bacterial pellets processed for ICP-MS analysis. The data shown are the means ±SD of intrabacterial zinc concentration expressed as nanograms Zn per grams of bacterial extract in one representative experiment performed in duplicate, out of two independent experiments. (B) Distribution of zinc crystals within macrophages and intraphagosomal bacilli. At selected times postinfection (p.i.) with *M. tuberculosis*, macrophages were treated for capturing of zinc ions by the AMG technique. Cells were then processed for EM observation. The zinc crystals formed during this procedure appeared as small dense deposits. Panels 1–3, intracellular localization of zinc crystals. The zinc crystals accumulated within mitochondria (M), along the inner face of the nuclear membrane (NMb) over the dense chromatin (panel 1), within lysosomes (L) but not in the Golgi (G) (panel 2), and also along the plasma membrane (PM) and the inner face of the phagosome membrane (PhM) (panel 3). Panels 4–7, localization of zinc crystals in intraphagosomal mycobacteria. Zinc was concentrated in three distinct parts of the *Mycobacterium*, i.e., on the outer surface (arrows in panel 4), in between the electron translucent layer of the cell wall and the cytoplasmic membrane (arrows in panel 5), and in the cytoplasm (arrows, panels 6 and 7) of live (panel 6) and dead (panel 7) bacilli. In panels 5 and 6, zinc crystals are also observed on the outer surface of the *Mycobacterium*. Scale bar, 0.5 μm. (C) Zinc distribution in wild-type and *ctpC* null mutant *M. tuberculosis* strains harbored in human macrophage phagosomes. Human macrophages were infected with either wild-type *M. tuberculosis* or its *ctpC* null mutant counterpart. At 4 and 24 hr p.i., cells were processed as in (B). Bacilli were scored for the presence of zinc at either the outer surface, the cell wall, or the cytoplasm. Quantitations were made on 100–150 bacilli per sample and in two independent experiments. Shown is the mean percent of zinc precipitate localization in one representative out of two experiments.

The striking induction of *ctpC* expression upon exposure of the bacteria to Zn^2+^ (Figure 3A), and its putative role as a zinc efflux pump, led us to consider it as an ideal mycobacterial gene candidate leading the resistance to zinc poisoning in human macrophages. To investigate its role as such, we used allelic exchange to construct a mutant of *M. tuberculosis* with an inactivated *ctpC* gene (Figures S3A and S3B). Upon direct exposure to zinc, the *ctpC* null mutant accumulated zinc as much as three times compared to the wild-type strain (Figure 4A). Yet, this susceptibility was specific only to zinc, since the mutant and wild-type strains contained to a similar degree equal amounts of other divalent cations such as Ni^2+^, Cu^2+^, and Cd^2+^ (Figure S3C).

To gain insight into the precise location of free zinc within intracellular *M. tuberculosis* or the *ctpC* null mutant, electron microscopy approaches were used. Because free zinc ions are too small to be visualized after fixation and processing by conventional methods (Figure S4A), an autometallographic (AMG) technique, adapted from Stoltenberg et al. (Stoltenberg et al., 2005) for capturing zinc ions, was applied to infected macrophages. Fixation with glutaraldehyde in the presence of sodium sulfide induces the formation of electron-dense zinc-sulfur nanocrystals that are subsequently silver enhanced by AMG. As long as the AMG development proceeds, the nanocrystals will grow in size. We have used a development time such that the nanocrystals reached 5–10 nm in size and were hence visible under the electron microscope, where they appeared as dense circular deposits (Figure S4B). The only disadvantage of this method, with respect to conventional EM methods, is that the cell ultrastructure and more especially the membrane of the different cellular organelles are less well preserved and, therefore, most of the time difficult to visualize (Figure S4B). It was clear, however, from control infected cells fixed in parallel by a conventional EM method that all the organelles were membrane bound and that all the bacilli were enclosed in membrane-bound phagosomes (Figure S4A). After infection with *M. tuberculosis*, the zinc crystals were observed within mitochondria and on the inner face of the nuclear membrane (Figures 4B, panel 1), within endocytic compartments (mostly lysosomes, Figures 4B, panel 2), and along the inner face of the plasma membrane (Figure 4B, panel 3). In addition, the inner face of the phagosome membrane was also studded with zinc crystals (Figure 4B, panel 3). We then examined individual bacilli for the presence or absence of zinc crystals (Figure 4B, panels 4–7). Three cases were distinguished in terms of location of zinc crystals, i.e., (1) exclusively along the outermost surface of the mycobacterial cell wall as in Figure 4B, panel 4; (2) within the cell wall as in Figure 4B, panel 5; and (3) within the cytoplasm (Figure 4B, panel 6). In the case of morphologically intact and live bacilli, the zinc crystals were in most cases restricted to the outer surface of the mycobacterial wall, as in Figure 4B, panel 4. In contrast, when bacteria were damaged and, therefore, dead, the cytoplasm usually contained zinc crystals (Figure 4B, panel 7). Based on these criteria, striking differences were revealed between wild-type *M. tuberculosis* and its *ctpC* null mutant counterpart (Figure 4C). In the case of the wild-type strain, zinc was localized mostly along the surface and within the cell wall in at least 93% of the morphologically intact bacilli, while some was found within the cytoplasm in as much as 7% within 24 hr (postinfection, p.i.). By contrast, the *ctpC* null mutant exhibited zinc within the cytoplasm in as much as 50% of the intact bacilli during the same time period (Figure 4C). Of note, while the percentage of damaged bacilli (as judged at the ultrastructural level) was identical (10%–12%) between the two strains during the first 4 hr (p.i.), it increased (up to 30%) only in the *ctpC* null mutant after 24 hr (p.i.).

**Figure 5:**
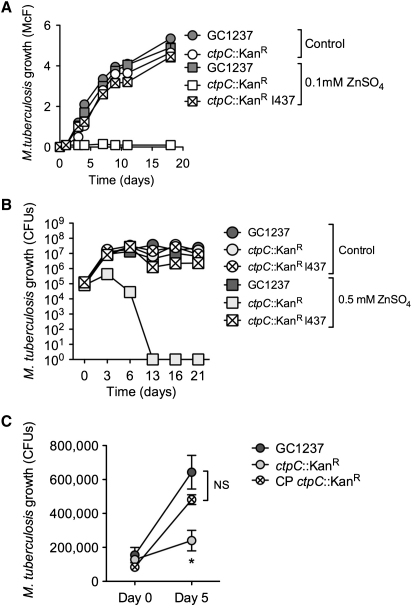
CtpC Is Involved in Zinc Detoxification and Contributes to the Intracellular Survival of *M. tuberculosis* (A) Differential sensitivity of *M. tuberculosis* wild-type and the *ctpC* null mutant to free zinc. *M. tuberculosis* wild-type (GC1237), a *ctpC* null mutant (*ctpC*::Kan^R^), or the cosmid-complemented strain (I437) was allowed to grow in 7H9-ADC medium containing 0.1 mM ZnSO_4_ or without zinc supplementation (Control). Bacterial growth was monitored by turbidity measurement (McFarland units). The data are representative of three independent experiments. (B) Differential sensitivity of *M. tuberculosis* wild-type and the *ctpC* null mutant to free zinc. *M. tuberculosis* wild-type (GC1237), a *ctpC* null mutant (*ctpC*::Kan^R^), or the cosmid-complemented strain (I437) were allowed to grow in 7H9-ADC medium containing 0.5 mM ZnSO_4_ or without zinc supplementation (Control). Bacterial growth was monitored by plating on agar and counting CFU. The data are representative of two independent experiments. (C) Differential ability of *M. tuberculosis* wild-type and the *ctpC* null mutant to replicate in human macrophages. *M. tuberculosis* wild-type (GC1237), a *ctpC* null mutant (*ctpC*::Kan^R^), or a plasmid-complemented strain (CP) was used to infect human macrophages at a multiplicity of infection of one mycobacteria per ten cells. After 4 hr, cells were washed and incubated in fresh medium for 5 days. The data shown are means ±SD of intracellular CFU counts in an experiment carried out in triplicate and analyzed with Student's t test. ^∗^p < 0.05; NS, not significant. The data are representative of three independent experiments.

Given this correlation between the accumulation of zinc and the morphological appearance of damaged bacilli, we then decided to test the ability for the *ctpC* null mutant to grow under different concentrations of zinc. As shown in Figures 5A and 5B and Figure S5A, the mutant was highly sensitive to zinc, as assessed by turbidity and counts of colony-forming units. Equally important, the wild-type phenotype was restored by genetic complementation with a cosmid encompassing the *ctpC* region of the genome, indicating the importance for this gene to resist zinc poisoning (Figures 5A and 5B). These observations also held true in the context of a macrophage infection. Although the phagosome containing both the wild-type and *ctpC* null strains accumulated zinc (Figure S5B), only the *ctpC* null mutant grew poorly in human macrophages, a deficiency that was soon restored upon expression of *ctpC* in the complemented strain (Figure 5C). To further investigate the role of CtpC in survival in vivo, we conducted infection experiments in immuno-competent or -deficient animals. However, we did not observe signs of attenuation of the *ctpC* null strain (Figure S6), indicating that there might be a compensation effect by other Ctp proteins among other reasons we later discuss below.

Nevertheless, our data provide compelling evidence that CtpC plays a crucial role in the ability of *M. tuberculosis* to resist zinc poisoning, at least in the context of human macrophages.

### Zinc Poisoning Contributes to the Killing of Nonpathogenic Bacteria by Macrophages

**Figure 6:**
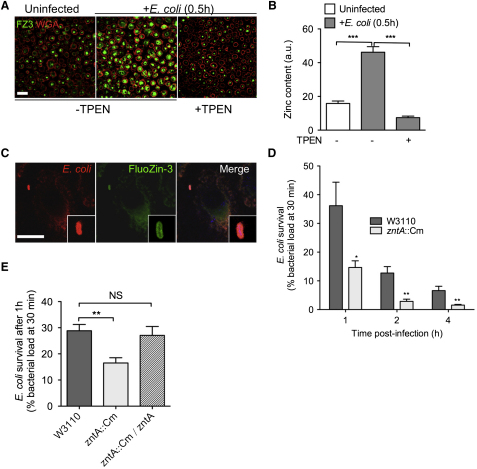
Zinc Accumulates in Classical Phagolysosomes and Contributes to Pathogen Killing (A) Labeling of free zinc in macrophages. Human macrophages were left uninfected (left panel) or infected for 30 min with *E. coli* (W3110) at a multiplicity of infection of five bacteria per cell (middle and right panels), in the absence (left and middle panels) or presence (right panel) of the zinc-chelating agent TPEN. Cells were fixed and stained with the zinc-specific fluorescent probe FluoZin-3 (FZ3, green), and the plasma membrane was stained with the lectin marker WGA (red). Scale bar, 40 μm. (B) FZ3 signal quantification (in arbitrary units) from ∼25 cells randomly chosen from three fields as in (A). Data are shown as in Figure 2B. (C) FZ3 staining of an *E. coli*-containing phagolysosome. A macrophage infected with Crimson-expressing *E. coli* (red), fixed and stained with FZ3, is shown. Scale bar, 10 μm. (D and E) Differential killing of *E. coli* wild-type and the *zntA* null mutant (D) or together with the complemented strain (*zntA*::Cm/*zntA*, E) by human macrophages. *E. coli* wild-type (W3110) or a *zntA* null mutant (*zntA*::Cm) was used to infect human macrophages at a multiplicity of infection of one bacterium per cell. After 0.5 hr, cells were washed and further incubated in fresh medium. After 1, 2, and 4 hr of infection, cells were lysed and cell lysates were plated onto agar for CFU scoring. The data shown are the means ±SD of intracellular *E. coli* CFU ratios, relative to CFU contents at 0.5 hr, in a triplicate experiment analyzed with Student's t test. ^∗^p < 0.05; ^∗∗^p < 0.01. All experiments were performed independently at least two times, and a representative experiment is shown.

It is clear from recent studies that host cells alter lysosomal and cytoplasmic zinc levels in response to bacterial pathogens or stimuli (Kitamura et al., 2006). While the impact due to these alterations in zinc concentrations is beginning to be understood in the context of immune cell differentiation and maturation (Kitamura et al., 2006; Murakami and Hirano, 2008), the impact on invading pathogens is thought to be solely growth hindrance due to zinc starvation, as predicted by the NRAMP model (Kehl-Fie and Skaar, 2010). Our results demonstrate that *M. tuberculosis* is subjected to zinc poisoning as an alternative use of this transition metal by human macrophages. Zinc poisoning may be a general feature of the innate immunity system. As zinc is freely released in late endosomes and lysosomes, to even higher levels than in the early endosomal compartment, we hypothesized that nonpathogenic microbes that are readily killed by macrophages might become even more susceptible in the absence of their zinc efflux systems. We tested this hypothesis by infecting human macrophages with either a nonpathogenic strain of *E. coli* or an *E. coli* mutant with an inactivated P_1B_-type zinc efflux ATPase ZntA (Helbig et al., 2008a, 2008b). As observed with *M. tuberculosis*, *E. coli* infection led to a burst of free zinc in infected macrophages (Figures 6A and 6B), some of which reached the *E. coli* phagolysosomes (Figure 6C). In addition, live imaging of FluoZin-3 in infected cells revealed FZ3-positive vesicular structures trafficking to and contacting FZ3-positive phagosomes (Movie S1). As predicted, the *zntA* null mutant of *E. coli* was killed faster than the wild-type strain by human macrophages (Figure 6D), and a wild-type phenotype was restored by genetic complementation (Figure 6E). Thus, zinc accumulation in phagolysosomes contributes to microbial control by macrophages.

## Discussion

This study illustrates how a parallel transcriptional survey between an intracellular microbe and its host cell may provide substantial insights into microbe physiology and host defenses. In the case of *M. tuberculosis*, our transcriptional analysis showed that, when engulfed by human macrophages, the bacillus faces a burst of free zinc, released in both early and late endosomal compartments, which reaches the mycobacterial phagosome. Such waves of free zinc are not observed in the presence of an inhibitor of the NADPH-dependent phagocyte oxidase, the enzyme involved in generating oxygen species. The zinc released in the cell therefore probably originates from the dissociation of intracellular Zn^2+^-MT complexes under oxidative conditions. MTs are thought to be mostly cytosolic proteins in eukaryotic cells (Maret, 2000), and the pumping of zinc into intracellular vesicles after its dissociation from Zn^2+^-MT complexes has been proposed as a general mechanism of cell protection from zinc toxicity (Lichten and Cousins, 2009). This raises questions about the way in which free zinc is transported into the endosomal-lysosomal pathway. The human genome encodes ten zinc transporters (ZnT1-10/SLC30A1-10) capable of transporting zinc to the outside of the cell across the plasma membrane (ZnT1/SLC30A1) or in vesicles (Lichten and Cousins, 2009). It remains unclear whether and which ZnTs are involved in zinc accumulation in the endosomal-lysosomal pathway in macrophages upon phagocytosis.

Our results are consistent with those of an earlier study in which the concentrations of several metal ions were measured in mycobacterial vacuoles in murine macrophages (Wagner et al., 2005). The authors of this study reported an increase in phagosomal zinc concentration by a factor of almost 10, from 37 to 459 μM, between 1 and 24 hr postinfection. This increase would be sufficient to induce *ctpC* transcription in *M. tuberculosis*, as we have shown in this study. In the presence of excess free zinc, only a limited number of *M. tuberculosis* genes are induced, essentially the putative P_1B_-type ATPase-encoding gene *ctpC* and its cognate putative metallochaperone-encoding gene *Rv3269*. We have not formally demonstrated that CtpC is a zinc efflux pump in *M. tuberculosis*, but this appears to be the case, since (1) it is phylogenetically related to zinc P_1B_-type ATPases, (2) its expression is induced upon exposure of bacilli to zinc, (3) a *ctpC* null mutant of *M. tuberculosis* accumulates zinc in a specific and uncontrolled manner, and (4) this mutant is highly sensitive to zinc. The exact mechanism by which the CtpC/Rv3269 couple may export zinc is currently under study. In fact, CtpC is required for the intracellular replication of *M. tuberculosis*, and the exact mode of mycobacterial killing by free zinc remains unknown. Although essential at low concentrations, zinc, like other heavy metals, is known to be toxic at high concentrations and to have pleiotropic effects on living organisms (Plum et al., 2010). For example, it is possible that, at high concentrations, zinc replaces other metal ions, such as copper, in a number of essential enzymes, leading to bacterial cell death. Zinc may also impair bacterial ATP generation by inhibiting the activity of cytochromes (Beard et al., 1995). Although we have not formally demonstrated that zinc uptake is required for zinc to be toxic to bacteria, it is worth noting that bacterial death correlates with increased cytosolic free zinc in our experiments (Figures 4 and 5). The exact mechanism(s) of zinc penetration into the bacteria, and of bacterial killing by free zinc, still remains to be fully understood.

In vivo experiments in immuno-competent or -deficient animals did not allow us to show signs of attenuation for the *ctpC* null mutant (Figure S6). This result was somehow surprising, and to a certain extent disappointing, but we think that there may be reasons for that. First, some compensation mechanism, for instance mediated by other Ctp proteins, may couteract *ctpC* deficiency; this may be addressed using mutants inactivated in several P-type ATPases, wich is ongoing in the laboratory. Second, we have used here high-dose challenge models (with inoculum sizes of up to 5000 CFUs). We may be able to detect an attenuation phenotype for the *ctpC* mutant in low-dose models (50–100 CFUs), or in other animal models, which is also ongoing in our laboratory. Last, it must be underlined here that zinc trafficking inside eukaryotic cells may be highly dependent on the cell type under consideration. For instance, TCR activation of T lymphocytes has been reported to induce zinc efflux from lysosomal storage compartments through the zinc transporter ZIP8/SLC39A8 (Aydemir et al., 2009), and extracellular zinc influx through the zinc transporter ZIP6/SLC39A6 (Yu et al., 2011); likewise, TLR4 stimulation of dendritic cells has been reported to decrease cytosolic free zinc, and affect their maturation (Kitamura et al., 2006). By contrast, FcR-mediated activation of mast cells has been reported to induce a burst or “wave” of free zinc in the cytosol, and this was shown to influence the ability of these cells to secrete cytokines (Yamasaki et al., 2007). In this context, understanding the mechanism of zinc release in macrophages, and whether this influences the immune capacities of these cells, may reveal aspects of the cell biology of zinc. In addition, this may explain the discrepancy between our results in vitro with human macrophages, and our results in vivo in mice.

The intracellular release of free zinc is not restricted to the phagocytosis of mycobacteria, as it also occurs when macrophages engulf other microbes, such as *E. coli*. In this case, zinc accumulates in maturing phagolysosomes, probably contributing to microbial killing because a mutant of *E. coli* with an inactivation of the well-characterized zinc efflux P_1B_-type ATPase ZntA is killed faster in macrophages than its wild-type counterpart. These results suggest that microbial poisoning by free zinc may constitute a mechanism used by macrophages, and possibly other phagocytes, to constrain microbes after their phagocytosis, thereby facilitating their clearance.

The intracellular poisoning of microbes by heavy metals in immune cells may also involve other ions, such as copper. One recent report showed that the copper transporter ATP7A mediates intracellular killing of *E. coli* (White et al., 2009). Intracellular poisoning with free copper may also occur in mycobacterial infections, because *ctpV*, a putative copper exporter-encoding gene, is induced in intracellular *M. tuberculosis* (Graham and Clark-Curtiss, 1999; Tailleux et al., 2008), and because *M. tuberculosis* mutants with inactivations of CtpV or of the putative outer membrane copper channel MctB/Rv1698 display altered virulence patterns in vivo (Ward et al., 2010; Wolschendorf et al., 2011). It remains to be evaluated whether and how free copper can reach microbial vacuoles within macrophages.

Our findings may have various implications for treatment. First, CtpC and other P-type ATPases, along with their putative functional partners, are potentially interesting targets for discovering treatments against mycobacteria and other microbes. Second, in the event that there is compensation between different Ctp proteins, *M. tuberculosis* strains deficient in multiple P-type ATPases may be attenuated enough to constitute promising live vaccine candidates for use as replacements for BCG. Third, understanding zinc homeostasis on a cell type per cell type basis may provide means to manipulate the overall immune system response for the benefit of the patient.

Rather than to serve as a contradiction to the NRAMP model that predicts vertebrate exploitation of bacterial requirement for transition metals (e.g., iron) by sequestering these elements, we propose zinc intoxication as an alternative and dynamic use of this metal by host cells, which may be tailored for the benefit of the patient. Indeed, our results and working model raise the question of micronutrient supplementation in patients with TB or other infections. Several clinical trials have been conducted to evaluate whether zinc supplementation, generally in conjunction with vitamin A, can help to accelerate classical antituberculous drug treatments. These studies have generated conflicting results (Green et al., 2005; Karyadi et al., 2002). The beneficial effect of zinc supplementation may be apparent only in individuals with low serum zinc concentrations, or may depend on the individual genetic background concerning zinc metabolism.

In conclusion, our results suggest that an intact zinc resistance system is necessary, but not sufficient, for intracellular parasitism by pathogenic mycobacteria. The presence of the *ctpC* gene in all mycobacteria, including harmless environmental species, strongly suggests that CtpC may represent a model example of so-called “exaptation,” as defined by S.J. Gould (Gould and Vrba, 1982), with *M. tuberculosis* using an ancient system of resistance to environmental metal poisoning present in its numerous soil-dwelling ancestors as an incidental solution to the modern problem of intracellular survival in eukaryotic phagocytes. Additional studies are required to improve our understanding of the role of zinc and other heavy metals in innate immunity to mycobacteria and other microbes, and to evaluate the extent to which the manipulation of such sophisticated cellular mechanisms of antimicrobial defense can be transferred into clinical practice for the benefit of patients and the community.

## Experimental Procedures

### Cells and Bacteria

Peripheral blood mononuclear cells (PBMCs) were isolated by centrifugation on a Ficoll gradient, washed, counted, and dispensed into 24-well plates in RPMI-1640 medium (GIBCO, Invitrogen) at a density of 2 × 10^6^ cells/well. Monocytes were purified by cell adherence to the plastic after 24 hr at 37°C and were incubated for 6 days in RPMI-1640 supplemented with 10% human AB serum and 20 ng/ml human macrophage colony-stimulating factor (M-CSF, Miltenyi Biotec) for differentiation into macrophages.

Mycobacteria were grown in Middlebrook 7H9 culture medium (Difco) supplemented with 10% albumin-dextrose-catalase (ADC, Difco), 0.05% Tween-80 (Sigma) in Sauton's medium supplemented with 0.05% Tween 80, or on Middlebrook 7H11 agar (Difco) supplemented with 10% oleic acid-ADC (OADC, Difco). See the Supplemental Experimental Procedures for more details.

### Construction of the *M. tuberculosis ctpC* Mutant and Complemented Strains

A *ctpC/Rv3270* mutant of *M. tuberculosis* GC1237 (Caminero et al., 2001) containing a disrupted *ctpC*::Kan^R^ allele was constructed by allelic exchange using the pPR23 plasmid, according to a procedure described elsewhere (Pelicic et al., 1997). See the Supplemental Experimental Procedures for more details.

### Metal Quantification

Total zinc, copper, nickel, and cadmium were quantified in dried bacterial pellets by inductively coupled plasma mass spectrometry (ICP-MS, Antellis, Toulouse, France). Briefly, samples (∼0.3 g) were mineralized in teflon vials with pure HNO_3_ (67%–69%) and H_2_O_2_ (36%) at 90°C for 24 hr. After dilution, samples were analyzed by ICP-MS (serie X2; Thermo Electron). The isotopes used in this study were as follows: ^66^Zn and ^68^Zn for total zinc quantification, ^63^Cu and ^65^Cu for total copper quantification, ^60^Ni and ^62^Ni for total nickel quantification, and ^111^Cd and ^114^Cd for total cadmium quantification. The four-point calibration curves used for quantification were obtained by dilution of a certified reference material (Inorganic Ventures). Detection limits of the method were 20 ng/g for Zn, 2 ng/g for Cu, 0.5 ng/g for Ni, and 0.05 ng/g for Cd.

### Macrophage Transfection

We used a Neon MP 5000 electroporation system (Invitrogen) for transient expression of RFP-tagged proteins in human macrophages. The experiments were performed in accordance with the manufacturer's instructions, with the following parameters: 1000 V, 40 ms, two pulses, and 1 μg of DNA for 2 × 10^5^ cells. Cells were used within 24 hr of transfection.

### Confocal Microscopy

For visualization of free zinc, fixed cells were incubated for 1 hr with the cell permeant reagent FZ3 (Invitrogen) at a final concentration of 1 μM in PBS. Images were observed with a LSM710 microscope equipped with a 40× 1.30 NA objective (Carl Zeiss, Inc.), recorded with Zen software (Carl Zeiss, Inc.), and analyzed with ImageJ software. See the Supplemental Experimental Procedures for more details.

### AMG Silver Enhancement and Processing for Electron Microscopy

The procedure as described here is a modification of the method described by Stoltenberg et al. (Stoltenberg et al., 2005) that had been used for 1–2 mm thick slices of tissue. See the Supplemental Experimental Procedures for more details.

### Mouse Infection

Six- to eight-week-old female Balb/c or SCID mice were anesthetized with a cocktail of ketamine (100 mg/kg; Merial) and xylasine (15 mg/kg; Bayer) and infected intranasally with ∼5000 CFUs of the various mycobacterial strains (see text) in 25 μl of saline/0.01% Tween 80. After 1, 21, and 42 days, Balb/c mice were sacrificed by cervical dislocation, and lung homogenates were plated onto agar for CFU scoring. See the Supplemental Experimental Procedures for more details.

### qPCR and Microarray Analysis

Eukaryotic RNA was extracted with the RNeasy mini kit (QIAGEN). Mycobacterial RNA was extracted with a modified version of the RNeasy mini kit protocol. For RT-qPCR, cDNA was synthesized with the SuperScript First-Strand Synthesis System Kit (Invitrogen) and random hexamer primers. We performed qPCR in a final volume of 25 μl, with a sequence detection system (model ABI Prism 7300; Applied Biosystems) and Power SYBR-Green PCR mastermix (Applied Biosystems). See the Supplemental Experimental Procedures for more details.

### Statistical Analysis

All experiments performed at least in triplicate were analyzed using the Student's t test, eventually corrected (Welch's formula) when variances differed between the two samples, according to the F test.

## Supplemental Information

Document S1. Six Figures, Two Tables, Supplemental Experimental Procedures, and Supplemental References

Movie S1. Time-Lapse Fluorescence Microscopy of FZ3 Staining of *E. coli* Vacuoles in a Human Macrophage Human macrophages were infected at a moi of 10, stained without fixation with FZ3, and observed lived by confocal microscopy. Arrowheads point to FZ3 vesicular structures contacting FZ3-positive phagosomes. Accelerated 75 times.
