# Supplementary material for: Mycobacterial P1-Type ATPases Mediate Resistance to Zinc Poisoning in Human Macrophages
Source: Cell Host Microbe. 2011 Sep 15;10(3):248–59. doi: 10.1016/j.chom.2011.08.006 (PMC3221041; doi:10.1016/j.chom.2011.08.006)
Supplement: Document S1. Six Figures, Two Tables, Supplemental Experimental Procedures, and Supplemental References [file mmc1.pdf]

Cell Host & Microbe, Volume *10*

Supplemental Information

Mycobacterial P_1-Type ATPases Mediate Resistance to Zinc Poisoning in Human Macrophages

Hélène Botella, Pascale Peyron, Florence Levillain, Renaud Poincloux, Yannick Poquet, Irène Brandli, Chuan Wang, Ludovic Tailleux, Sylvain Tilleul, Guillaume Charrière, Simon J. Waddell, Maria Foti, Geanncarlo Lugo-Villarino, Qian Gao, Isabelle Maridonneau-Parini, Philip D. Butcher, Paola Ricciardi Castagnoli, Brigitte Gicquel, Chantal de Chastellier, and Olivier Neyrolles

INVENTORY OF SUPPLEMENTAL INFORMATION

Figure S1. The expression of several *M. tuberculosis* metal cation-transporting P-type ATPases and related genes is induced in host phagocytes

Figure S2. FZ3 staining of *M. tuberculosis*-infected macrophages

Figure S3. Generation and characterization of the *ctpC*-null mutant

Figure S4. Morphological appearance of, and zinc distribution in human macrophages infected with *M. tuberculosis*.

Figure S5. Additional phenotypic characterization of the *M. tuberculosis ctpC*-null mutant.

Figure S6. The *ctpC*-null mutant is not attenuated *in vivo* in mice.

Table S1. Significant features of the zinc stress response following exposure of *M. tuberculosis* to high concentrations of zinc (0.5 mM), as revealed by microarray analysis

Table S2. List of oligonucleotides used in the study

Movie S1. Time-lapse fluorescence microscopy of FZ3 staining of *E. coli* vacuoles in a human macrophage.

Supplemental Experimental Procedures

Supplemental References

SUPPLEMENTAL FIGURES

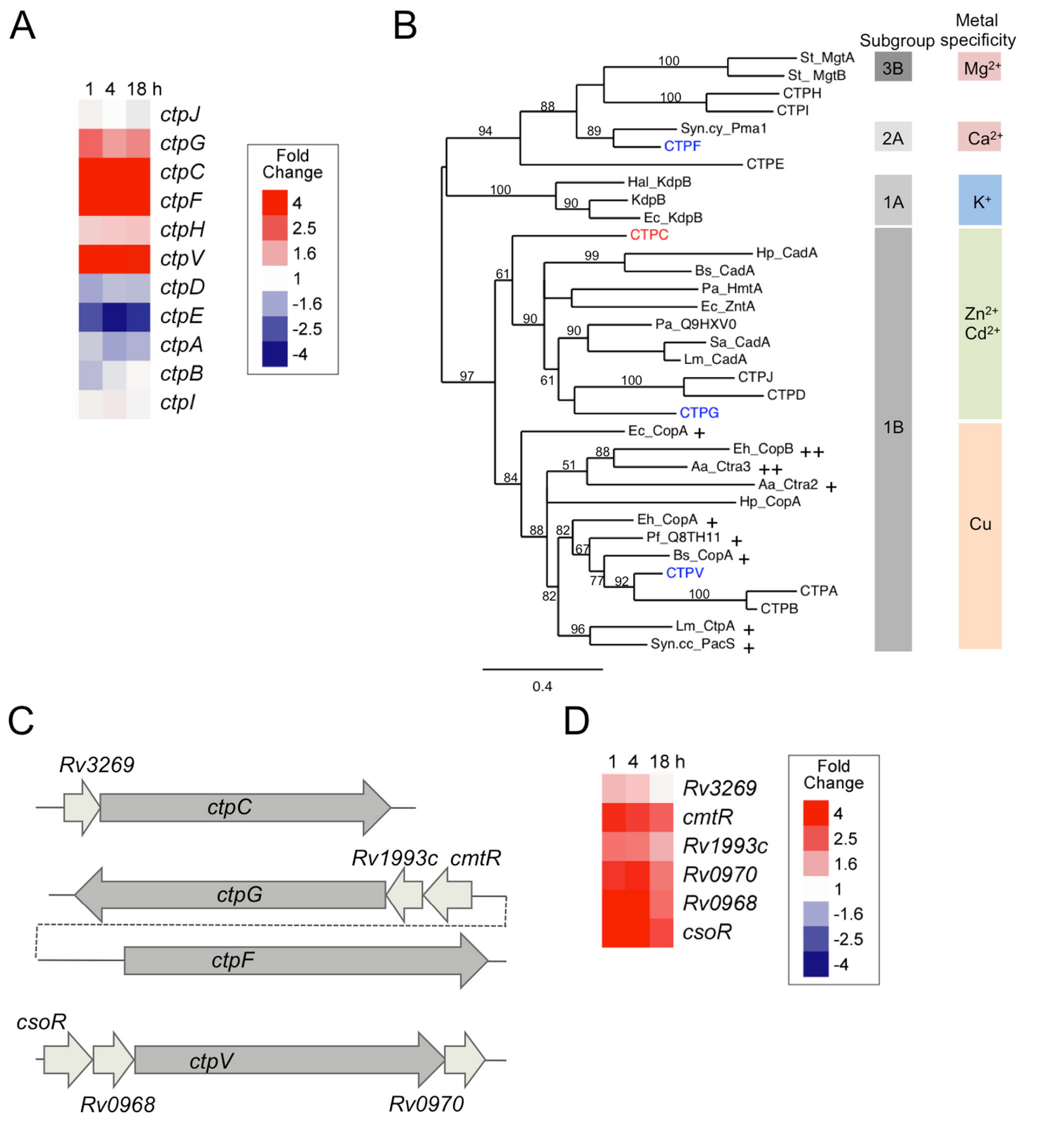

Figure S1. The expression of several *M. tuberculosis* metal cation-transporting P-type ATPases and related genes is induced in host phagocytes

(A) Transcriptional profile analysis. Red-blue density display showing the expression ratio of *M. tuberculosis* metal cation-transporting P-type ATPase-encoding genes (*ctpA-J* & *ctpV*) at 1, 4 or 18 h in macrophages relative to aerobic growth *in vitro*, as described in microarray

analysis by Tailleux *et al.* (Tailleux *et al.*, 2008). Genes are ordered in rows, conditions as columns. Red indicates genes induced in intracellular conditions with respect to aerobic growth conditions (fold change); blue indicates repression in intracellular conditions.

(B) Molecular phylogeny of the P-type ATPases across *M. tuberculosis* and 23 other bacterial species. The molecular phylogeny of all predicted *M. tuberculosis* P-type ATPases and of 23 well characterized P-type ATPases from other bacterial species was explored with the Phylogeny.fr interface (Dereeper *et al.* 2008 & Dereeper *et al.* 2010). Subgroups and metal specificity are indicated as in a previous study (Arguello *et al.* 2007). St, *Salmonella typhimurium*; Syn.cy, *Synechocystis*; Hal, *Halobacterium*; Ec, *Escherichia coli*; Hp, *Helicobacter pylori*; Bs, *Bacillus subtilis*; Pa, *Pseudomonas aeruginosa*; Sa, *Staphylococcus aureus*; Lm, *Listeria monocytogenes*; Eh, *Enterococcus hirae*; Aa, *Aquifex aeolicus*; Pf, *Pyrococcus furiosus*; Syn.cc, *Synechococcus*.

(C) Illustration depicting the genetic organization of the *M. tuberculosis* metal cation-transporting P-type ATPase-encoding genes *ctpC* (Rv3270), *ctpG* (Rv1992c), *ctpF* (Rv1997) and *ctpV* (Rv0969). Genes encoding putative metal chaperones (Rv3269, Rv1993c and Rv0968) and metal-responsive transcriptional regulators (cmtR/Rv1994c and csoR/Rv0967) are shown.

(D) Transcriptional profile analysis. Red-blue density display showing the expression ratio of *M. tuberculosis* genes related to the metal cation-transporting P-type ATPase-encoding genes *ctpC*, *ctpG* and *ctpV* at 1, 4 or 18 h in and macrophages relative to aerobic growth *in vitro*, as described in microarray analysis by Tailleux *et al.* (Tailleux *et al.*, 2008). Genes are ordered in rows, conditions as columns. Red indicates genes induced in intracellular conditions relative to aerobic growth conditions (fold change); blue indicates repression.

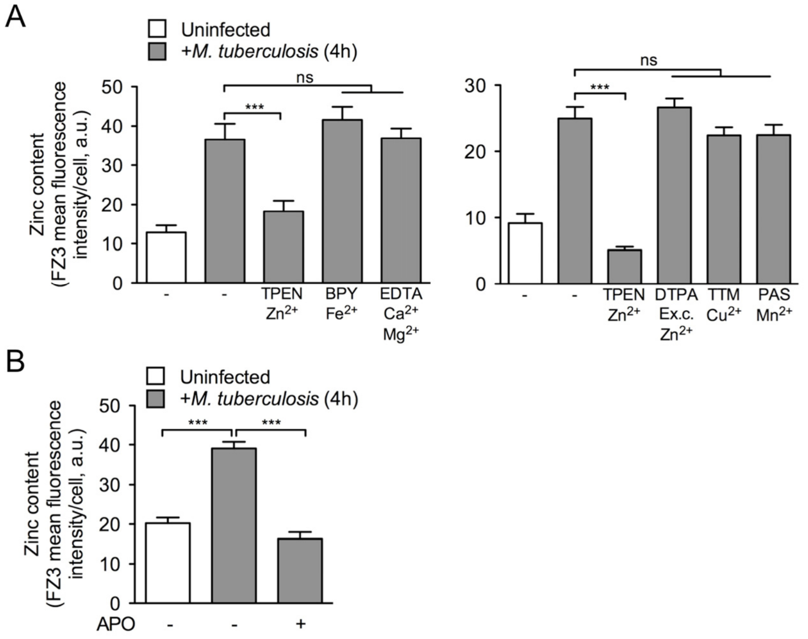

Figure S2. FZ3 staining of *M. tuberculosis*-infected macrophages

(A) Quantification (in arbitrary units) of the FZ3 signal of human macrophages before and after *M. tuberculosis* infection, in the presence of various chelators. Human monocyte-derived macrophages were infected for 4 h with *M. tuberculosis* at a multiplicity of infection of 5 mycobacteria per cell in the presence of 20 μM zinc-, iron-, calcium/magnesium-, extracellular zinc-, copper- or manganese-chelating agents (TPEN, BPY, EDTA, DTPA, TTM or PAS, respectively). After infection, cells were fixed and stained with FZ3. The data show means±s.d. of the FZ3 signal measured from ~20 cells and were analyzed with Student's *t*-test. ***, $P<0.001$; ns, not significant.

(B) Quantification (in arbitrary units, as in A) of the FZ3 signal of human macrophages before and after *M. tuberculosis* infection, in the presence of the NADPH-oxidase inihibitor apocynin (APO).

Experiments shown in (A) and (B) are representatives of at least two independent experiments.

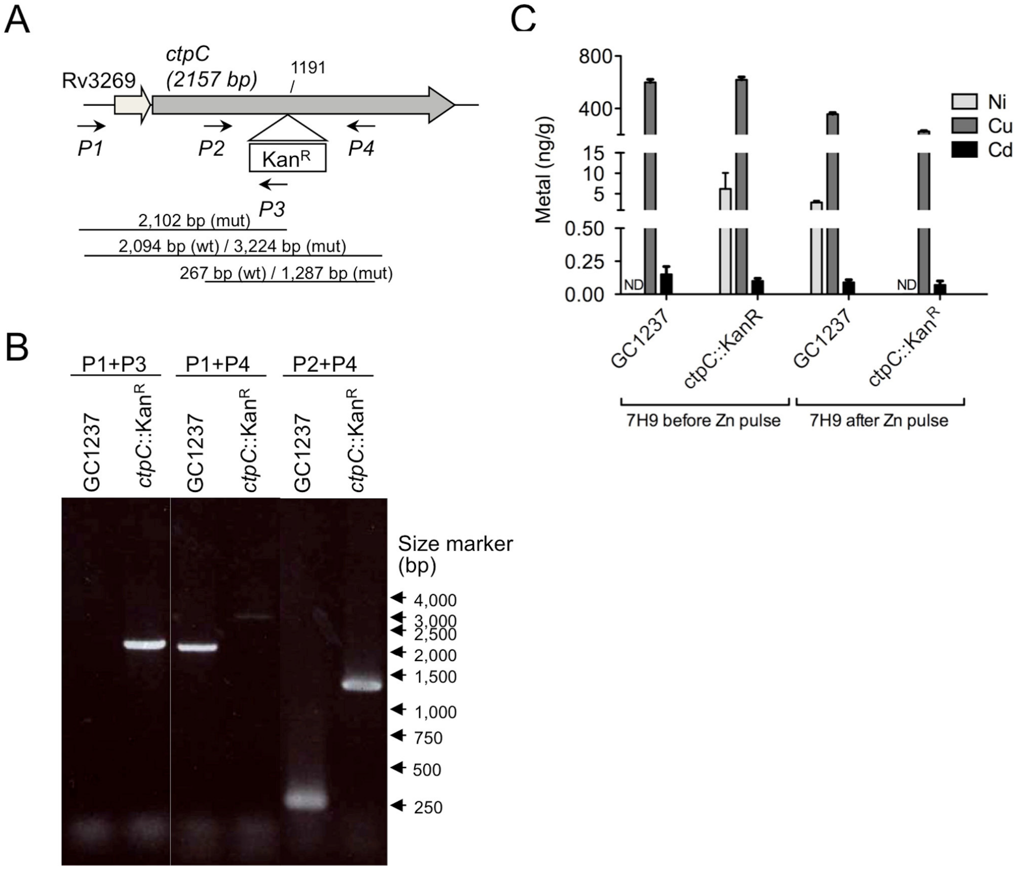

Figure S3. Generation and characterization of the *ctpC*-null mutant

(A) A schematic representation of the genetic organization of the putative Rv3269-*ctpC* operon. The position of the oligonucleotides (P1-4) used for PCR validation and the expected size of the resulting amplicons are shown.

(B) PCR analysis of the *ctpC*-null mutant, with the primers shown in (A).

(C) Metal content of the *ctpC*-null mutant. *M. tuberculosis* wild-type (GC1237) or the *ctpC*-null mutant (*ctpC* ::Kan^R) was left untreated (before Zn pulse) or incubated with 0.1 mM $ZnSO_4$ (after Zn pulse) for 1 h at 37°C, washed twice in PBS, heat-inactivated and the bacterial pellets were processed for ICP-MS analysis. The data shown are the means±s.d. of intrabacterial nickel, copper and cadmium concentrations (ng/g) in one experiment performed in duplicate.

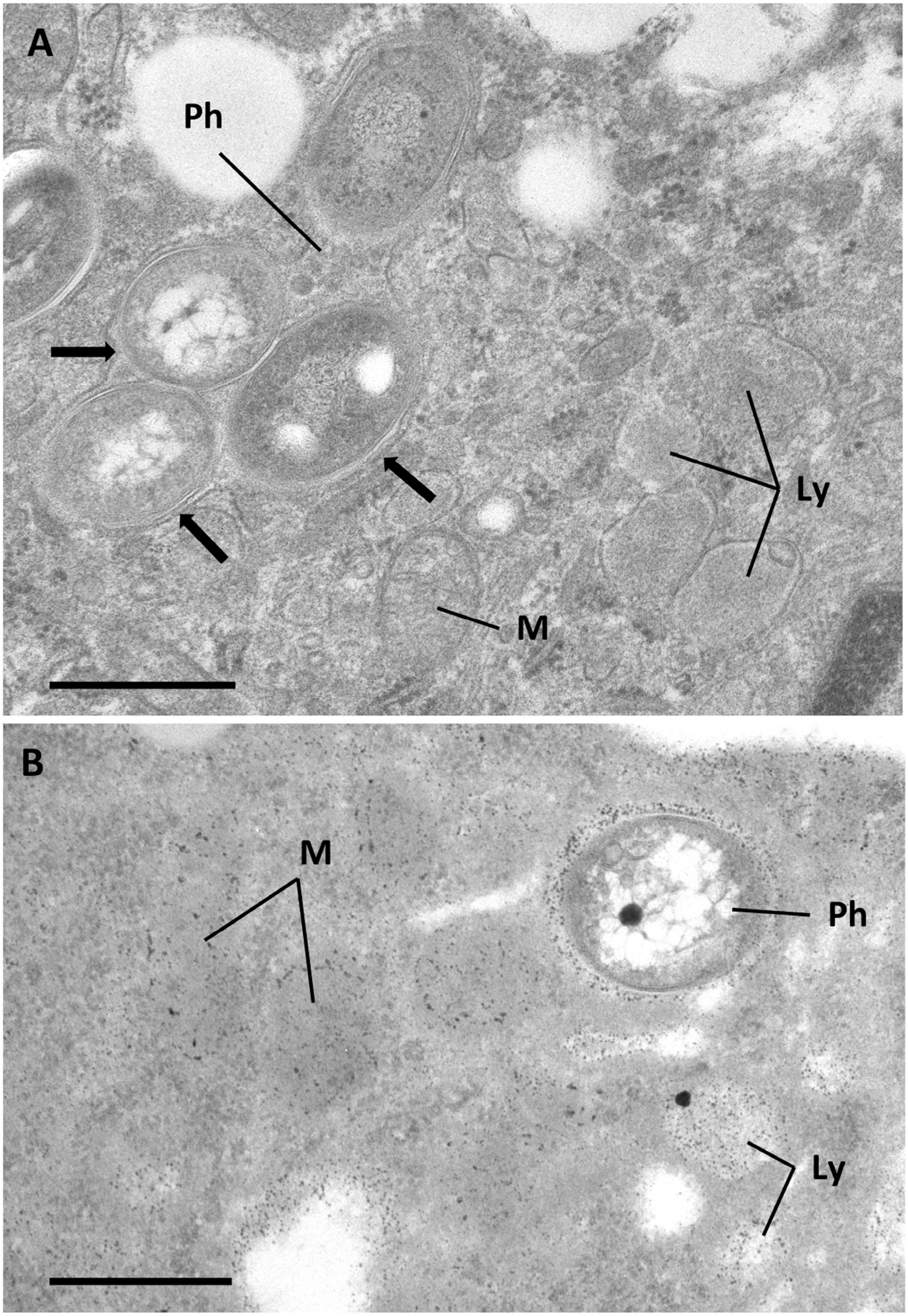
A
Ph
Ly
M
B
M
Ph
Ly

Figure S4. Morphological appearance of, and zinc distribution in human macrophages infected with *M. tuberculosis*. Thin sections of **human macrophages** infected with *M. tb* were processed at 4hr p.i. either according to a conventional EM procedure (A) or to the AMG procedure (B). A) In control cells fixed according to the normal procedure for conventional electron microscopy, free zinc ions are too small to be visualized. As a consequence the organelles cited above are all free of electron dense deposits. In contrast, the fixation conditions are such that all the membranes can be well visualized. In such conditions, all the bacilli were found to reside within membrane-bound phagosomes (arrows). B) Free intracellular zinc ions were captured by fixing cells with 2.5% glutaraldehyde in presence of 0.1% sodium sulfide. This induced the formation of zinc sulfur nanocrystals that were further enhanced with silver by the AMG method. On this general view, the small dense precipitates corresponding to the free zinc ions formed during this procedure can be seen within mitochondria (M), lysosomes (Ly) and also in an *M. tb*-containing phagosome (Ph) in which they are located between the outer surface of the mycobacterium and the phagosome membrane. Due to the AMG procedure, the cell ultrastructure, and more especially the membrane of the different cellular organelles, including phagosomes, lacks definition, and is, therefore, usually difficult to visualize. Scale bar= 0.5μm.

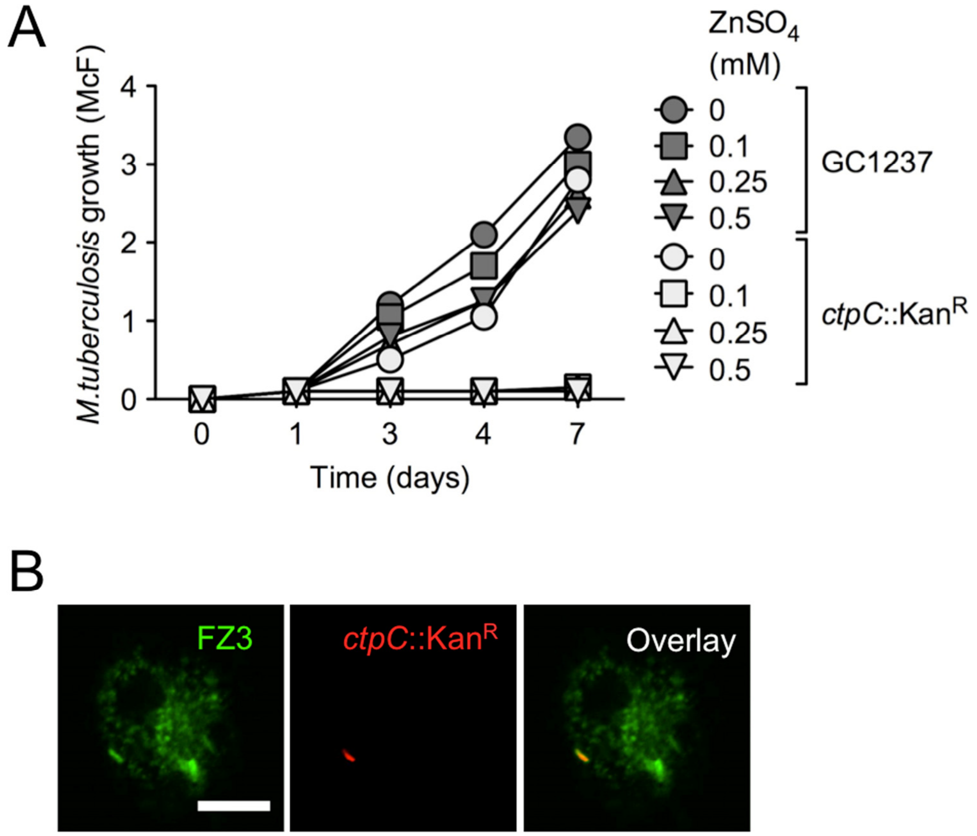

Figure S5. Additional phenotypic characterization of the *M. tuberculosis ctpC*-null mutant.

(A) Differential sensitivity of *M. tuberculosis* wild-type and the *ctpC*-null mutant to free zinc. *M. tuberculosis* wild-type (GC1237) or the *ctpC*-null mutant (*ctpC* ::Kan^R) was allowed to grow in 7H9-ADC medium containing 0.1, 0.25 or 0.5 mM $ZnSO_4$, or without zinc supplementation (Control). At various times, bacterial growth was monitored by turbidity measurement (McFarland units).

(B) FZ3 staining of the *M. tuberculosis ctpC*-null mutant phagosome, and analysis by confocal microscopy. One macrophage infected with the *M. tuberculosis ctpC*-null mutant stained with TRITC (red), fixed and stained with FZ3 (green) is shown. Scale bar, 10 µm.

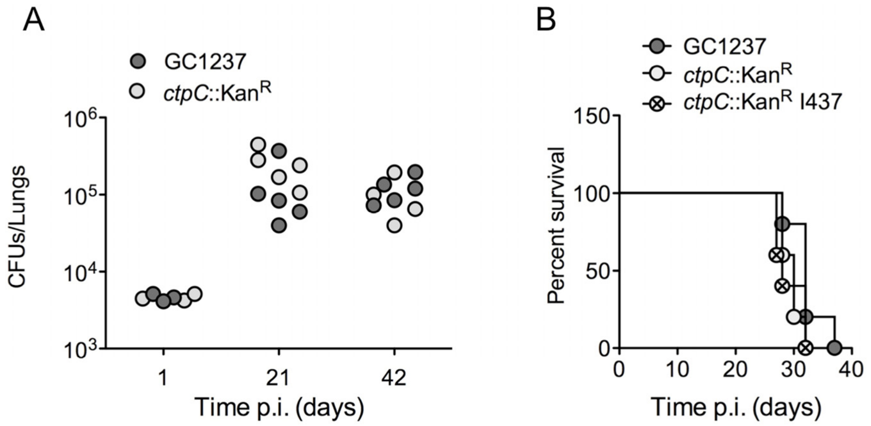

Figure S6. The *ctpC*-null mutant is not attenuated *in vivo* in mice.

(A) Balb/c mice were infected intranasally with ~5,000 CFUs of *M. tuberculosis* wild-type (GC1237) or the *ctpC*-null mutant. At day 1, 21 and 42 after infection, mice were sacrificed and lung homogenates were plated onto agar for CFU scoring. Each circle corresponds to one animal.

(B) Immuno-deficient SCID mice were infected intranasally with ~5,000 CFUs of *M. tuberculosis* wild-type (GC1237), the *ctpC*-null mutant or its complemented strain, and survival was monitored over time. Data shown in (A) and (B) correspond to one representative experiment out of two independent experiments.

LEGEND TO SUPPLEMENTAL MOVIE

Movie S1. Time-lapse fluorescence microscopy of FZ3 staining of *E. coli* vacuoles in a human macrophage.

Human macrophages were infected at a MOI of 10, stained without fixation with FZ3, and observed lived by confocal microscopy. Arrowheads point to FZ3 vesicular structures contacting FZ3-positive phagosomes. Accelerated 75 times.

SUPPLEMENTAL TABLES

Table S1. Significant features of the zinc stress response following exposure of *M. tuberculosis* to high concentrations of zinc (0.5 mM), as revealed by microarray analysis

Gene name	Fold change (Zn *vs*. no Zn)[1]	*P* value	Putative function[2]
cadI	5.51	0.043	Cadmium-inducible protein
ctpC	4.42	0.028	Metal cation-transporting P-type ATPase
Rv2642	4.13	0.043	Transcriptional regulator, arsR-family
Rv3269	3.83	0.055	Conserved hypothetical protein
Rv1993c	3.82	0.043	Conserved hypothetical protein
arsC	3.38	0.043	Arsenic-transporting membrane protein
cysK2	3.12	0.029	Cysteine synthase A
ctpG	2.18	0.095	Metal cation-transporting P-type ATPase
PPE27	0.50	0.024	PPE family protein
tpx	0.49	0.094	Thiol peroxidase
accA3	0.49	0.023	Bifunctional acetyl-/propionyl-coenzyme A carboxylase alpha chain
Rv0799c	0.47	0.060	Conserved hypothetical protein
gltA1	0.47	0.024	Citrate synthase I
Rv2160c	0.44	0.091	Conserved hypothetical protein
TB18.6	0.44	0.091	Conserved hypothetical protein
Rv3847	0.43	0.014	Hypothetical protein
Rv0760c	0.42	0.091	Conserved hypothetical protein
Rv0079	0.42	0.091	Hypothetical protein
Rv2005c	0.42	0.043	Conserved hypothetical protein
mmpL3	0.40	0.100	Transmembrane transport protein
thiD	0.39	0.029	Phosphomethylpyrimidine kinase
Rv3127	0.32	0.014	Conserved hypothetical protein
Rv2626c	0.32	0.060	Conserved hypothetical protein
Rv0097	0.31	0.024	Oxidoreductase
Rv1738	0.27	0.034	Conserved hypothetical protein
esxD	0.18	0.014	Esat-6-like protein

1. Only genes with a fold change >2 or <0.5 are shown.

2. Function is given according to TubercuList (http://genolist.pasteur.fr/TubercuList/).

Table S2. List of oligonucleotides used in the study

Name	sequence
ctpCKOFd	5'-GTTTAAACTCGTTGGTGCAGGTGGCCAATC-3'
ctpCKORv	5'-GTTTAAACTCGGATCCGTCGCGTTGAC-3'
P1	5'- TCGGCGTCACCGACTATGCGACC-3'
P2	5'- GCGCGCGATGACCATGTTGTTG -3'
P3	5'-ATCGCGGCCTCGAGCAAGACG-3'
P4	5'- CTGGCGGCATAGGCCAGCACT-3'
ctpA-Fd	5'-CACCGTCAAACTCAACATGG-3'
ctpA-Rv	5'-CAAGCTGTTTGAGACCACGA-3'
ctpB-Fd	5'-TGTGACCGTCTTCGTTCTTG-3'
ctpB-Rv	5'-CTCGCCGGTATCACTAGCTC-3'
ctpC-Fd	5'-TCACCATTTTCACCGGGTAT-3'
ctpC-Rv	5'-'GATGTTGAGCAACCACAGGA-3'
ctpD-Fd	5'-CCGAGCAGAAGGTTGAAGTC-3'
ctpD-Rv	5'-AGTTCGTCCCGTATGGTCAC-3'
ctpE-Fd	5'-CCGTGAAACGCTGGATTATT-3'
ctpE-Rv	5'-ACGGCCAAAACTGGTGTAAG-3'
ctpF-Fd	5'-CCGAACTCGGTGAGATTCAT-3'
ctpF-Rv	5'-GATGGCGATGGTCAGAAACT-3'
ctpG-Fd	5'-AGTTCACCGAGGTCATCGTC-3'
ctpG-Rv	5'-AGTCCGATCAGGTGTCTTGG-3'
ctpH-Fd	5'-GGTGCTCACCGGAAACTCTA-3'
ctpH-Rv	5'-ACTTCGATGTACCTCGGCTG-3'
ctpI-Fd	5'-CTGTCCTACGAACCGGTGAT-3'
ctpI-Rv	5'-AGTAGCGCGTCGATATTGCT-3'
ctpJ-Fd	5'-GATGACCTGACCACCATTCC-3'
ctpJ-Rv	5'-CATTGAGTCCGACGATGATG-3'
ctpV-Fd	5'-GCAGGACAAGGTAGCTGAGG-3'
ctpV-Rv	5'-GACATTAGCGTGATGTCGGA-3'
Rv3269-Fd	5'- TGGCGATACAAGTGTTCTTGGCG-3'
Rv3269-Rv	5'-ACGTCGGCCACCTTTAGGCGG-3'
SigA-Fd	5'-CTCGACGCTGAACCAGACCT-3'
SigA-Rv	5'-AGGTCTTCGTGGTCTTCGTC-3'
MTF1-Fd	5'-ATTGAAGGTGCAACCCTCACT-3'
MTF1-Rv	5'-CAAAGGTGTACTCTCCTCGGT-3'
MT1-Fd	5'-ATGGACCCCAACTGCTCCTG-3'
MT1-Rv	5'-TCAGTCGCAGCAGCTGCAC-3'
MT2-Fd	5'-ATGGATCCCAACTGCTCCTG-3'
MT2-Rv	5'-TCAGGCGCAGCAGCTGCAC-3'
Znt1-Fd	5'-CCCCGCAGACCCAGAAAAC-3'
Znt1-Rv	5'-GTTGTCCAGCCCTATCTTCTTC-3'
HPRT-Fd	5'-CTCATGGACTGATTATGGACAGG-3'
HPRT-Rv	5'-GCTGACCTGCTGGATTACATTAA-3'

SUPPLEMENTAL EXPERIMENTAL PROCEDURES

Cells and bacteria

Peripheral blood mononuclear cells (PBMCs) were obtained from healthy blood donors (*Etablissement Français du Sang*, EFS, Toulouse). Written informed consent to obtained human blood was obtained from the donors, under EFS contract no. 21/PVNT/TOU/IPBS01/2009-0052. In accordance with articles L1243-4 and R1243-61 of the French Public Health Code, the contract was approved by the French Ministry of Science and Technology (agreement no. AC 2009-921).

The *M. tuberculosis ctpC* mutant was grown in the presence of 25 µg/ml kanamycin. The complemented mutant strains were grown in the presence of 25 µg/ml hygromycin plus kanamycin. *E. coli* strains were grown in LB medium. The *E. coli zntA* mutant and complemented strain (Helbig *et al.*, 2008a; Helbig *et al.*, 2008b) were obtained from Dr D.H. Nies (Molecular Microbiology, Institute for Biology/Microbiology, Martin-Luther-University, Halle-Wittenberg, Germany), and were grown in the presence of chloramphenicol (20 µg/ml) or ampicillin (100 µg/ml) and chloramphenicol, respectively.

Plasmids and reagents

The plasmids encoding mRFP-Rab5 and Lamp1-RFP were obtained from Addgene; the plasmid encoding cathepsin D-RFP was kindly provided by Dr. F. Darchen (Institut de Biologie Physico-Chimique, Paris, France). The pE2-Crimson plasmid was purchased from Clontech. All the following reagents were purchased from Sigma-Aldrich and used as indicated in the text: N,N,N',N'-Tetrakis(2-pyridylmethyl)ethylenediamine (TPEN), 4-aminosalicylic acid (PAS), diethylenetriaminepentaacetic acid (DTPA), ammonium tetrathiomolybdate (TTM), 2,2'-bipyridyl (BPY), ethylenediaminetetraacetic acid (EDTA), apocynin.

Construction of the *M. tuberculosis ctpC* mutant and complemented strains

Briefly, a DNA fragment overlapping the *ctpC* gene was amplified by PCR from genomic DNA with the oligonucleotides ctpCKOFd and ctpCKORv (Table S2). It was inserted, after the insertion of a pACYC177 (New England Biolabs)-derived kanamycin resistance cassette (Kan^R), into the unique *Xho*I restriction site of pPR23. The resulting plasmid was transferred by electroporation into *M. tuberculosis*, and an allelic exchange at the *ctpC* locus was screened by PCR analysis of the genomic DNA from several kanamycin- and sucrose-resistant colonies, with the following primers: P1, P2, P3 and P4 (Table S2 and Figure S3A,B). One *M. tuberculosis* clone with a pattern corresponding to the disruption of *ctpC* was

selected and named *ctpC*::Kan^R. A complemented strain was obtained by electroporation of the *ctpC*::Kan^R mutant with a pYUB412-derived cosmid (Bange *et al.*, 1999), MTCI437, which carries a 38-kb DNA fragment covering the 3645-3683 kb genomic region of *M. tuberculosis* H37Rv and the hygromycin resistance cassette. Another complemented strain was obtained by cloning *ctpC* in the plasmid pMIP12 (Le Dantec *et al.*, 2001) carrying the hygromycin resistance cassette instead of the kanamycine resistance cassette. For TRITC staining of the bacteria, *M. tuberculosis ctpC*-null mutant was incubated in 0.01% tetramethylrhodamine isothiocyanate (TRITC, Sigma) in 0.1 M sodium carbonate buffer (pH 9), 150 mM NaCl, during 30 minutes at room temperature, then washed three times in PBS and used for macrophages infection.

Macrophage infection

For infection, macrophages were incubated with bacteria in RPMI-10% AB serum at various multiplicities of infection and for various times (see text). For bacterial survival experiments, infected cells were washed after 4 h (mycobacteria) or 30 min (*E. coli*) with phosphate-buffered saline (PBS, without Ca^{2+} or Mg^{2+}, GIBCO Invitrogen) to remove extracellular bacteria, and were then incubated in fresh medium. At the indicated time points, cells were lysed with 500 µl of 0.1% Triton X-100 in sterile water, and viable intracellular bacteria were counted by plating serial dilutions of the lysates onto Middlebrook 7H11 agar-10% OADC (mycobacteria) or LB-agar (*E. coli*) medium. For confocal microscopy experiments, after the indicated time, cells were washed with PBS and fixed by incubation in 4% methanol-free paraformaldehyde (PFA, Polysciences Inc.) in PBS for 1 hour at room temperature.

Confocal microscopy

For MTF1 labeling, cells were permeabilized by incubation with 0.2% saponin in PBS for 5 minutes, then incubated with a primary antibody against MTF1 (SantaCruz Biotechnology) in 0.05% saponin-2% fetal bovine serum (FBS) in PBS for 1 h, then washed 3 times in PBS. Cells were then incubated with a goat anti-rabbit IgG (H+L) secondary antibody coupled to Alexa Fluor 488 (Invitrogen), and with TO-PRO-3 iodide (Invitrogen) for 1 hour in 0.05% saponin-2% FBS in PBS for nuclear staining. Cells were then washed 3 times in PBS. In some experiments, cells were incubated with Alexa-594 wheat germ agglutinin (WGA, Invitrogen) to stain plasma membranes. After labeling, coverslips were set in Fluoromount G (SouthernBiotech) on microscope slides.

Time-Lapse Fluorescence Microscopy Analysis

Human macrophages were cultivated in 8-well Lab-Tek Chamber Slide System (Thermo Scientific). Cells were infected for 20 min at 37°C with Crimson-expressing *E. coli* at a MOI of 10 bacteria/cell, washed in PBS, and incubated for 20 min at 37°C with 5 µM FZ3 in Hepes buffer. Cells were then washed in PBS and observed in a 37°C temperature-controlled box in Hepes buffer. Confocal microscopy analysis was performed with a LSM710 microscope equipped with a x40 1.30 NA objective (Carl Zeiss, Inc.), recorded with Zen software (Carl Zeiss, Inc.) and movie was mounted with ImageJ software. Images were acquired every 15 s.

AMG silver enhancement and processing for electron microscopy

At selected time points p.i., cells were fixed overnight at 4°C with 2.5% glutaraldehyde in 0.1M Na-cacodylate buffer containing 0.1M sucrose and 0.1% sodium sulfide. Cells were thoroughly washed with sucrose-containing Na-cacodylate buffer, and incubated for 60 min at RT, in the dark, with 1.5 ml of AMG developer. The latter consisted of a 60 ml gum Arabic solution and 10 ml Na-citrate buffer (25.5 g citric acid, 1 H_2O + 23.5 g Na-citrate, 2 H_2O added to 100 ml distilled water), 15 ml reductor (0.85 g of hydroquinone dissolved in 15 ml distilled water at 40°C) and 15 ml of a solution containing silver ions (0.12 g silver lactate in 15 ml distilled water at 40°C) which were added immediately before use, while thoroughly stirring the AMG solution (Danscher, 1981). The AMG development was stopped by replacing the developer with a 5% Na-thiosulfate solution (in distilled water) for 10 min. Cells were washed extensively with distilled water (5 successive baths for 1 min each) and further processed for electron microscopy. After fixation and treatment according to the AMG procedure described above, cells were post-fixed for 1h at RT with 1% osmium tetroxide in 0.1M Na-cacodylate buffer, pH 7.2, devoid of sucrose. They were washed with buffer, scraped off the dishes, concentrated in 2% agar in cacodylate buffer and treated for 1 h at room temperature with 1% uranyl acetate in Veronal buffer. Samples were dehydrated in a graded series of ethanol and embedded in Spurr resin. Thin sections (70nm-thick) were stained with 1% uranyl acetate in distilled water and then with lead citrate. As a control, infected cells were processed for conventional electron microscopy. In this case, cells were first fixed for 1h at RT with 2.5% glutaraldehyde in 0.1M Na-cacodylate buffer, pH 7.2, containing 0.1M sucrose, 5mM $CaCl_2$ and 5mM $MgCl_2$ before fixation with osmium tetroxide and subsequent processing as indicated above above. For quantification, 100-150 different

bacilli per sample were examined for the distribution of zinc crystals on the outer surface of bacilli, the cell wall and the cytoplasm. Care was taken to avoid serial sections.

Mouse infection

Mice were housed in pathogen-free conditions and treated according to institutional animal care protocol no. 20080318/9, approved by the Regional Ethics Committee of Midi-Pyrénées (France) for Animal Experimentation (authorization no. MP/01/36/06/08). The survival of SCID mice was monitored over time, and mice were sacrificed when they showed signs of suffering.

qPCR and microarray analysis

Briefly, the bacterial pellet was resuspended in 700 µl of RLT buffer supplemented with 1% β-mercaptoethanol and 500 µl of 0.1-mm glass beads (Biospec). Bacteria were disrupted with a bead beater (Qbiogene) for 5 minutes at 30 $m.s^{-1}$. After centrifugation for 15 s at 10,000x*g*, the supernatant was transferred to a new microcentrifuge tube and the beads were washed with an additional 300 µl of 1% β-mercaptoethanol-RLT buffer for 1 minute in the bead beater. After centrifugation for 15 s at 10,000x*g*, the supernatants were combined and treated according to the manufacturer's recommendations (Qiagen). The residual genomic DNA was digested with an RNase-free DNase I (Ambion). RNA quality was assessed with a NanoDrop 1000 apparatus (NanoDrop).

The threshold cycle (Ct; the number of the cycle at which the amount of the amplified gene of interest reaches a fixed threshold) was then determined. Relative mRNA levels were calculated by the comparative Ct method. Relative levels of target mRNA, normalized with respect to an endogenous control (*sigA* for the mycobacterial RNAs, *HPRT* for the eukaryotic RNAs), were expressed as $2^{-\Delta\Delta Ct}$ (fold), where ΔCt = Ct of the target gene − Ct of the control gene (*sigA* or *HPRT*), and ΔΔCt = ΔCt of the studied set of conditions − ΔCt of the calibrator conditions, as previously described (Livak and Schmittgen, 2001). The following oligonucleotide pairs were used to detect *M. tuberculosis ctpA-J, ctpV, Rv3269* and *sigA*, respectively: ctpA-J-Fd and ctpA-J-Rv, ctpVFd and ctpVRv, Rv3269-Fd and Rv3269-Rv, SigA-Fd and SigA-Rv (Table S2). The following oligonucleotide pairs were used to detect *H. sapiens MTF1*, *MT1*, *MT2*, *ZNT1/SLC30A1* and *HPRT*, respectively : MTF1-Fd and MTF1-Rv, MT1-Fd and MT1-Rv, MT2-Fd and MT2-Rv, Znt1-Fd and Znt1-Rv, HPRT-Fd and HPRT-Rv (Table S2).

For microarray experiments to assess the effect of zinc on the mycobacterial transcriptome, mycobacteria were exposed to 0, 50 or 500 mM $ZnSO_4$ for four hours. RNAs were extracted

using the procedure described above, were checked with the Nanodrop ND 1000 and Bioanalyzer 2100 Expert (Agilent), and were labeled with the Quick Amp Labeling kit (Agilent) where the oligodTprimer was replaced by a random primer T7 (SBI system Bioscience). The labeled RNAs were hybridized to a customized *M. tuberculosis* pan-genomic microarray (Agilent, AMADID reference 019943) according to the One-Color Microarray-Based Prokaryote Analysis Protocol (Agilent). Microarray chips contained 60-mer oligonucleotide probes designed for 4,040 open reading frames and 1,670 intergenic regions in the genome of *M. tuberculosis* H37Rv. In average 3 probes were used for each ORF, and 1 probe in each orientation in intergenic regions. Non-mRNA (structural RNAs) transcripts were excluded. The hybridised slides were scanned on an Axon 4000B (Molecular devices) and analyzed with FeatureExtraction V.9 (Agilent). Data were pre-processed, normalized, and analyzed using the LIMMA (linear models for microarray analysis) library of the Bioconductor *R* package (Smyth, 2005).

Phylogenetic analysis

We used Muscle software to align the sequences. Ambiguous regions (i.e. containing gaps and/or poorly aligned) were removed with Gblocks. The phylogenetic tree was reconstructed by the maximum likelihood method, as implemented in the PhyML program, with the WAG matrix and a gamma correction for variable rates of evolution. Internal branch reliability was assessed with the aLRT test. The values reported are aLRT values and only aLRT values >50 are shown.

Microarray access

Fully annotated microarray data have been deposited in BμG@Sbase (accession number E-BUGS-122; http://bugs.sgul.ac.uk/E-BUGS-122) and also ArrayExpress (accession number E-BUGS-122).

SUPPLEMENTAL REFERENCES

Wagner, D., Maser, J., Lai, B., Cai, Z., Barry, C.E., 3rd, Honer Zu Bentrup, K., Russell, D.G., and Bermudez, L.E. (2005). Elemental analysis of *Mycobacterium avium*-, *Mycobacterium tuberculosis*-, and *Mycobacterium smegmatis*-containing phagosomes indicates pathogen-induced microenvironments within the host cell's endosomal system. J Immunol *174*, 1491-1500.

Ward, S.K., Abomoelak, B., Hoye, E.A., Steinberg, H., and Talaat, A.M. (2010). CtpV: a putative copper exporter required for full virulence of *Mycobacterium tuberculosis*. Mol Microbiol *77*, 1096-1110.

White, C., Lee, J., Kambe, T., Fritsche, K., and Petris, M.J. (2009). A role for the ATP7A copper-transporting ATPase in macrophage bactericidal activity. J Biol Chem *284*, 33949-33956.

Wolschendorf, F., Ackart, D., Shrestha, T.B., Hascall-Dove, L., Nolan, S., Lamichhane, G., Wang, Y., Bossmann, S.H., Basaraba, R.J., and Niederweis, M. (2011). Copper resistance is essential for virulence of *Mycobacterium tuberculosis*. Proc Natl Acad Sci U S A *108*, 1621-1626.

Yamasaki, S., Sakata-Sogawa, K., Hasegawa, A., Suzuki, T., Kabu, K., Sato, E., Kurosaki, T., Yamashita, S., Tokunaga, M., Nishida, K*., et al.* (2007). Zinc is a novel intracellular second messenger. J Cell Biol *177*, 637-645.

Yu, M., Lee, W.W., Tomar, D., Pryshchep, S., Czesnikiewicz-Guzik, M., Lamar, D.L., Li, G., Singh, K., Tian, L., Weyand, C.M*., et al.* (2011). Regulation of T cell receptor signaling by activation-induced zinc influx. J Exp Med *208*, 775-785.
