# Supplementary figures and images for: Mycobacterial P1-Type ATPases Mediate Resistance to Zinc Poisoning in Human Macrophages
Source: Cell Host Microbe. 2011 Sep 15;10(3):248–59. doi: 10.1016/j.chom.2011.08.006 (PMC3221041; doi:10.1016/j.chom.2011.08.006)

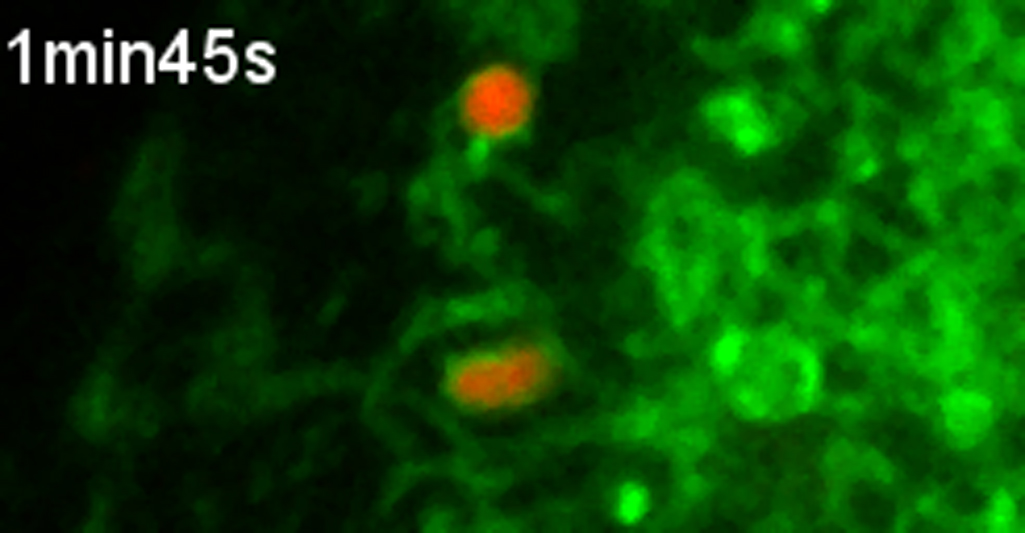

Supplement: Movie S1. Time-Lapse Fluorescence Microscopy of FZ3 Staining of E. coli Vacuoles in a Human Macrophage — Human macrophages were infected at a moi of 10, stained without fixation with FZ3, and observed lived by confocal microscopy. Arrowheads point to FZ3 vesicular structures contacting FZ3-positive phagosomes. Accelerated 75 times. [file mmc2.jpg]
